# Clinical and hematological characteristics of children infected with the omicron variant of SARS-CoV-2: role of the combination of the neutrophil: lymphocyte ratio and eosinophil count in distinguishing severe COVID-19

**DOI:** 10.3389/fped.2024.1305639

**Published:** 2024-06-24

**Authors:** Qiaoyan Jin, Wenxian Ma, Wei Zhang, Huiyuan Wang, Yiongxiang Geng, Yan Geng, Yang Zhang, Dan Gao, Jing Zhou, Lin Li, Yaping Gou, Bo Zhong, Jing Li, Wei Hou, Shemin Lu

**Affiliations:** ^1^ Department of Pediatrics, The Second Affiliated Hospital of Xi’an Jiaotong University, Xi’an, China; ^2^ Department of Biochemistry and Molecular Biology, Xi’an Jiaotong University Health Science Center, Xi’an, China; ^3^ Xijing 986 Hospital Department, Air Force Medical University, Xi’an, China; ^4^ Department of Cardiovascular Medicine, The Second Affiliated Hospital of Shaanxi University of Traditional Chinese Medicine, Xi’an, China; ^5^ National Joint Engineering Research Center of Biodiagnostics and Biotherapy, The Second Affiliated Hospital of Xi'an Jiaotong University, Xi’an, China

**Keywords:** COVID-19, children, neutrophil: lymphocyte ratio, eosinophil count, severe infection

## Abstract

**Purpose:**

Investigate the clinical/hematological characteristics of children infected with the Omicron variant of severe acute respiratory syndrome-coronavirus-2 (SARS-CoV-2) and identify an effective indicator to distinguish coronavirus disease 2019 (COVID-19) severity in children.

**Methods:**

A retrospective study was conducted through electronic medical records from pediatric patients. The demographic, clinical, and routine blood test (RBT) features of children diagnosed by real-time PCR for SARS-CoV-2 were collected.

**Results:**

Data of 261 patients were analyzed. The most common abnormality shown by RBTs was increased monocyte count (68%). Children had “mild-moderate” or “severe” forms of COVID-19. Prevalence of abnormal neutrophil count (*p* = 0.048), eosinophil count (*p* = 0.006), mean corpuscular volume (*p* = 0.033), mean platelet volume (*p* = 0.006), platelet-large cell ratio (*p* = 0.043), and red blood cell distribution width-standard deviation (*p* = 0.031) were significantly different in the two types. A combination of the neutrophil: lymphocyte ratio (NLR) and eosinophil count for diagnosing severe COVID-19 presented the largest AUC (0.688, 95% CI = 0.599–0.777; *p* < 0.001), and the AUC increased with a decrease in age.

**Conclusions:**

Combination of the NLR and eosinophil count might be a promising indicator for identifying severe COVID-19 in children at infection onset.

## Introduction

Severe acute respiratory syndrome-coronavirus-2 (SARS-CoV-2), like other viruses, evolves over time (1). Correspondingly, the properties of SARS-CoV-2, such as transmissibility, clinical characteristics, and severity, might vary with a new strain or variant. Five variants of concern have been identified by the WHO (1): Alpha, Beta, Gamma, Delta, and Omicron. Some mutations strengthen the fitness advantage of a variant, which makes the virus more infectious and transmissible. Epidemiological studies have revealed that the Delta variant spreads ∼60% faster than the Alpha variant (2). The Omicron variant has adaptive advantages over the Delta variant (2), and is transmitted more readily than the Delta variant. In addition, these mutations enable the virus to evade vaccine-mediated immunity (1, 2). Thus, the properties of SARS-CoV-2 mutations reiterate the importance of continued investigation of the infection features of each variant/strain to understand SARS-CoV-2 evolution. Relevant clinical data can also help to annotate the genomic sequences of new mutations.

Due to care within the home and fewer outdoor activities at the beginning of the coronavirus disease 2019 (COVID-19) pandemic, the incidence of COVID-19 was lower in children compared with that in adults (3, 4). In addition, an immature immune system (5), different features of angiotensin-converting enzyme-2 (6), fewer underlying abnormalities, and a healthier respiratory tract led to milder severity and a lower prevalence of mortality due to COVID-19 in children than in adults. However, with an increase in the transmissibility and virulence of new variants of SARS-CoV-2, the prevalence of infection (7, 8), COVID-19-caused hospitalizations (9), and severe complications (10) have increased in children. Moreover, with the elimination of isolation and quarantine policies, a higher risk of exposure to SARS-CoV-2 variants has emerged (8, 11). Thus, SARS-CoV-2 will continue to pose a threat to the overall health of children.

Nowadays, more patients with COVID-19 are seeking medical help in nearby primary care units or secondary care units. If medical resources are limited, some examinations related to COVID-19 severity (e.g., measurement of levels of proinflammatory cytokines) (12) cannot be carried out. Therefore, greater attention should be paid to: (i) analyze the features of children with severe COVID-19; (ii) explore a readily available and cost-effective indicator to reflect COVID-19 severity in children. This strategy could: (i) assist pediatricians to identify severe illness in the early stage and make targeted interventions; (ii) prevent the deterioration of infection and improve the prognosis of children with severe COVID-19.

We summarized the clinical and hematological features of children with SARS-CoV-2 infection in Xi'an, China, during an Omicron epidemic. We explored the identifying indicators of severe COVID-19 in children at an early stage. These findings could contribute to appropriate management to reduce the impact of COVID-19 on children worldwide.

## Methods and materials

### Ethical approval of the study protocol

The study protocol was approved by the ethics committee of the Second Affiliated Hospital of Xi'an Jiaotong University (Xi'an, China) and was undertaken in compliance with the Declaration of Helsinki 1964 and its later amendments. The parents or guardians of children provided verbal consent.

### Study design and patients

We conducted a retrospective study at the pediatric clinic or emergency department of the Second Affiliated Hospital of Xi'an Jiaotong University. The study period was from 11 to 31 December 2022. Following 1 week of implementation of the management strategy, patients suffering from COVID-19 could visit a specialist clinical department. With the consent of their parents or guardians, nasopharyngeal swabs were taken from children. These specimens underwent real-time reverse transcription-polymerase chain reaction (RT-PCR) to detect the RNA of SARS-CoV-2. The diagnostic definition of COVID-19 was established according to the *Diagnosis and Treatment Protocol for COVID-19 Patients (Trial Version 10)* (DTPC-10) issued by the National Health Commission of China (13). A positive result of RT-PCR was the primary criterion for a confirmed case of COVID-19. Children with COVID-19 aged <14 years who completed routine blood tests (RBTs) and whose medical record was available were enrolled in our study.

Most of the criteria for COVID-19 severity have been developed based on the degree of need for oxygen supplementation. The criteria used in DTPC-10 include a broad spectrum of symptoms, such as seizures and dehydration. Such criteria are in accordance with the current understanding that SARS-CoV-2 infection can cause functional impairment of multiple systems. More details of symptoms (e.g., duration and degree of fever) were included in our study. According to the protocol set in DTPC-10 (13), COVID-19 was classified into three types: “mild-to-moderate”, “severe”, and “critical” (Table 1).

**Table 1:** Criteria for the different types of severity of COVID-19 in children.

Severity	Details
Mild-to-moderate	The patient presents any of the following symptoms: Symptoms of infection of the upper respiratory, such as fever, sore throat, itchy throat, or cough Persistent high fever >3 days Tachypnea, respiratory rate (RR) < 30 bpm, and, in a resting state, oxygen saturation >93% on air inhalation Specific pneumonia manifestations of COVID-19 on imaging
Severe	The patient presents with any of the following symptoms: Ultra-hyperpyrexia Tachypnea (<2 months of age, RR ≥60 bpm; 2–12 months of age, RR ≥50 bpm; 1–5 years of age, RR ≥40 bpm; > 5 years of age, RR ≥ 30 bpm), except for the effects of fever and crying In a resting state, oxygen saturation ≤93% on air inhalation. Nasal ale flap, assisted respiration, wheeze or stridor Consciousness disorders or seizure Aphasia or feeding difficulties, dehydration
Critical	The patient presents with any of the following symptoms: Respiratory failure and the need for mechanical ventilation Shock Combined with other organ failure necessitating monitoring or treatment in the intensive care unit

### Laboratory detection

#### Routine blood tests (RBTs)

At the time of the first visit, blood specimens were taken from the peripheral veins of children. RBTs were undertaken using an automatic blood cell analyzer within an hour (catalog number: XN-9100-10B2; Sysmex, Shanghai, China). The neutrophil: lymphocyte ratio (NLR), monocyte: lymphocyte ratio (MLR), and platelet: lymphocyte ratio (PLR) was calculated.

#### Nucleic acids of SARSCoV-2

Real-time RT-PCR detected the open reading frame 1ab (Ct-ORF) and nucleocapsid protein (Ct-N) of *SARS-CoV-2* in nasopharyngeal swabs. The detection procedure followed the manufacturer's instructions for a SARS-CoV-2 nucleic acid detection kit (20221345; Daan Gene, Guangzhou, China). The cycle threshold (Ct) represents the viral load. Ct <40 denoted that the nucleic acids of SARS-CoV-2 were present in the nasopharyngeal swab.

### Data collection

We reviewed all confirmed cases of COVID-19. We collected data at the first hospital visit through electronic medical records using a structured questionnaire. We recorded: demographics (sex, age); date of the hospital visit; time interval from disease onset to the hospital visit; clinical symptoms; medical history; treatment strategy; Ct value of RT-PCR upon the diagnosis; and RBT data. These data profiles were reviewed independently by different researchers.

### Statistical analyses

The Kolmogorov–Smirnov test was applied to test for a variable distribution. Continuous variables are shown as the median [interquartile range (IQR)]. Comparisons between the two groups were made by the Mann–Whitney test. Categorical variables are shown as numbers and percentages. We undertook Fisher's exact test or the chi-square test to compare differences between the two groups. The Kruskal–Wallis test was employed for pairwise comparisons in multiple groups. Binary logistic regression analysis was carried out for parameters with differences between the mild-to-moderate group and the severe group. A receiver operating characteristic (ROC) curve was used to evaluate the prediction ability of the logistic regression model. The ROC curve and the area under the ROC (AUC) were used to evaluate the diagnostic benefit of single or combined hematological parameters for children suffering from severe COVID-19. The maximum value of the Youden Index was employed to estimate the sensitivity and specificity corresponding to the optimal cutoff value. *p *< 0.05 was considered significant. Statistical tests were undertaken using SPSS 16.0 (IBM, Armonk, NY, USA).

## Results

### Demographic and clinical characteristics of COVID-19 in children

The study population comprised 110 girls (42.1%) and 151 boys (52.9%) (Table 2). The median age was 2.0 (IQR, 0.65–7.0) years. Among the population, 71 (27.2%) were school-age (6–12 years), 47 (18.0%) were preschool-age (3–6 years), 76 (29.1%) were toddlers (1–3 years), 65 (24.9%) were infants (28 days to 1 year), and two (0.8) were newborns (<28 days). The median interval from symptom onset to the first hospital visit was 1.0 (1.0–2.0) day. The history of disease before COVID-19 included febrile seizures (FSs; 19, 7.28%), epilepsy (nine, 3.44%), asthma (three, 2.30%), acute laryngitis (two, 0.77%), nephrotic syndrome (19, 7.28%), and other (allergic rhinitis, congenital diaphragmatic hernia, anemia, hypothyroidism, autoimmune encephalitis, leukemia, myasthenia gravis; seven, 3.07%).

**Table 2:** Demographic and clinical characteristics of children with COVID-19.

Characteristics		Total (*n* = 261)	Mild-to-moderate (*n* = 210)	Severe (*n* = 51)	*p*
Sex, *n* (%)	Female	110 (42.1)	93 (44.3)	17 (33.3)	0.155
	Male	151 (57.9)	117 (55.7)	34 (66.7)	
Age, year (median, interquartile range)		2.0 (0.9–6.0)	2.0 (0.65–7.0)	2.0 (1.0–4.0)	0.565**
Age groups, *n* (%)	Newborn (≤28 days)	2 (0.8)	2 (1.0)	0 (0.0)	0.002
	Infant (<1 year)	65 (24.9)	60 (28.6)	5 (9.8)	
	Toddler (1–3 years)	76 (29.1)	52 (24.8)	24 (47.1)	
	Preschool-age (3–6 years)	47 (18.0)	34 (16.2)	13 (25.5)	
	School-age (6–14 years)	71 (27.2)	62 (29.5)	9 (17.6)	
Ct (mean ± SD) by RT-PCR	ORF1ab gene	30.61 ± 4.279	30.62 ± 4.460	30.55 ± 3.476	0.918*
	N gene	29.89 ± 4.407	29.91 ± 4.555	29.73 ± 3.776	0.795*
Time interval (median, interquartile range)		1.0 (1.0–2.0)	1.0 (1.0–2.0)	1.0 (0.5–2.0)	0.013**
Presence of underlying disease		42 (16.1)	21 (10.0)	21 (41.2)	<0.001
Category of underlying disease	Febrile seizures	20 (7.7)	9 (4.3)	11 (21.6)	<0.001
	Epilepsy	9 (3.4)	3 (1.4)	6(11.8)	<0.001
	Asthma	3 (1.1)	2 (1.0)	1 (2.0)	0.481
	Acute laryngitis	2 (0.8)	0 (0.0)	2 (3.9)	0.038
	Nephrotic syndrome	2 (0.8)	2 (1.0)	0 (0.0)	1.000
	Other	7 (2.7)	5 (2.4)	2 (3.9)	0.626
Treatment before visit		226 (86.6)	18 (86.7)	44 (86.3)	0.941
Drug categories	Antipyretics	187 (71.6)	147 (70.0)	40 (78.4)	0.457
	Antitussive	22 (8.4)	21 (10.0)	1 (2.0)	0.088
	Antiviral TCM	53 (20.2)	49 (23.3)	4 (7.8)	0.016
	Cold medicine	20 (7.7)	19 (9.0)	1 (2.0)	0.134
	Antibiotic	20 (7.7)	18 (8.6)	2 (3.9)	0.388
Clinical manifestation					
Fever		252 (96.6)	204 (97.1)	48 (94.1)	0.384
	Low-grade	10 (3.8)	7 (3.3)	3 (5.9)	
	Moderate	101 (38.7)	80 (38.1)	21 (41.2)	
	Hyperpyrexia	138 (52.9)	116 (55.2)	22 (43.1)	
	Ultra-hyperpyrexia	2 (0.8)	0 (0.0)	2 (3.9)	
Cough		144 (55.2)	129 (61.4)	15 (29.4)	<0.001
Runny nose		59 (22.6)	52 (24.8)	7 (13.7)	0.091
Expectoration		52 (19.9)	44 (21.0)	8 (15.7)	0.398
Nausea and emesis		45 (17.2)	37 (17.6)	8 (15.7)	0.838
Convulsive seizures		38 (14.6)	0 (0.0)	38 (74.5)	<0.001
Stuffy nose		26 (10.0)	25 (11.9)	1(2.0)	0.033
Hoarseness		23 (8.8)	19 (9.0)	4 (7.8)	1.000
Dry/itchy/sore throat		22 (8.4)	21 (10.0)	1 (2.0)	0.88
Rash		8 (3.1)	7 (3.3)	1 (2.0)	1.000
Abdominal pain		7 (2.7)	6 (2.9)	1 (2.0)	1.000
Headache		7 (2.7)	6 (2.9)	1 (2.0)	1.000
Wheeze/dyspnea		9 (3.4)	2 (1.0)	7 (13.7)	<0.001
Ache all over		5 (1.9)	4 (1.9)	1 (2.0)	1.000
Sneeze		4 (1.5)	3 (1.4)	1 (2.0)	0.583
Diarrhea		4 (1.5)	4 (1.9)	0 (0.0)	1.000
Appetite redaction		3 (1.1)	3 (1.4)	0 (0.0)	1.000
Allotriosmia		1 (0.4)	1 (0.5)	0 (0.0)	1.000
Conjunctival congestion		1 (0.4)	1 (0.5)	0 (0.0)	1.000
Complicated diagnosis					
Acute infection of the upper respiratory tract		218 (83.5)	177 (84.3)	41 (80.4)	0.501
Acute bronchitis		10 (3.8)	9 (4.3)	1 (2.0)	0.692
Acute suppurative tonsillitis		4 (1.5)	3 (1.4)	1 (2.0)	0.583
Acute laryngitis		20 (7.7)	15 (7.1)	5 (9.8)	0.557
Pneumonia inflammation		9 (3.4)	7 (3.3)	2 (3.9)	0.690
Herpetic pharyngotonsillitis		4 (1.5)	4 (1.9)	0 (0.0)	1.000
Convulsive seizures		38 (14.6)	0 (0.0)	38 (74.5)	<0.001
Others		11 (4.2)	8 (3.8)	3 (5.9)	0.454
Therapy					<0.001
Oral drugs as an outpatient		178 (68.2)	160 (76.2)	18 (35.3)	<0.001
Medication infusion or hospitalization		83 (31.8)	50 (23.8)	33 (64.7)	<0.001

The total percentage may not be equal to 100% due to rounding up.

RT-PCR, reverse transcription-polymerase chain reaction; Ct, cycle threshold; Time interval, time from symptom onset to first hospital visit (day); TCM, traditional Chinese medicine.

Other underlying disease: allergic rhinitis, congenital diaphragmatic hernia, anemia, hypothyroidism, autoimmune encephalitis, leukemia, myasthenia gravis.

Other complications: myocardial damage, acute gastroenteritis, acute urticaria, abdominal pain; convulsive seizure.

* Student's *t*-test.

** Mann–Whitney test.

Fever (252, 96.6%) was the most common symptom. Respiratory symptoms such as cough (144, 55.2%), runny nose (59, 22.6%), expectoration (52, 19.9%), “stuffy” nose (26, 10.0%), hoarseness (23, 8.8%), dry/itchy/sore throat (22, 8.4%), wheeze/dyspnea (nine, 3.4%), and sneeze (four, 1.5%) were relatively common. Convulsive seizures (38, 14.6%) were also common. Digestive symptoms such as nausea and emesis (45, 17.2%), abdominal pain (7,2.7%), and diarrhea (4,1.5%) were comparatively rare. Few children suffered from rash (eight, 3.1%), headache (seven, 2.7%), body aches (five, 1.9%), appetite reduction (three, 1.1%), allotriosmia (one, 0.4%), dysphoria (one, 0.4%), or conjunctival congestion (one, 0.4%). Urinary tract infection and parageusia were not observed (Table 2).

A total of 218 (83.52%) children were diagnosed with acute infection of the upper respiratory tract, 38 (14.6%) suffered convulsive seizure, 20 (7.66%) had acute laryngitis, 10 (3.83%) children suffered acute bronchitis, nine (3.45%) had inflammation due to pneumonia, four (1.53%) were diagnosed with herpetic pharyngotonsillitis, two (0.77%) had myocardial damage, one (0.4%) suffered acute gastroenteritis, and three (1.15%) had acute urticaria. The advice proffered at the time of the first hospital visit was oral medication (178, 68.2%) or medication infusion/hospitalization (83, 31.8%). All patients were of mild-to-moderate (210, 80.46%) or severe (51, 19.54%) types (Table 2).

Among the 51 severe cases, 66.7% of patients (34/51) were male, the average age was 3.14 years old, and 41.2% (21/51) children had underlying disease. 78.5% (38/51) children suffered with convulsive seizures. 23 cases were admitted to the inpatient department for further treatment, there was no case with Kawasaki disease or death. Regarding the results of RBTs, decreased eosinophil counts were found in 62.8% (27/51) of patients with severe infection.

A comparative analysis was undertaken to find differences in demographic and clinical characteristics between mild-to-moderate and severe types (Table 2). There were no differences in the ratio of sexes (*p *= 0.155) or age (*p *= 0.565) between the two types. The distribution of COVID-19 severity in children of different ages was significant (*p *= 0.002). The proportion of mild-to-moderate infections in infants was higher, and that of severe infections in toddlers and preschoolers was higher. Most (49/53, 92.5%) children who had taken a traditional Chinese medicine (TCM) formulation as an antiviral agent orally before the first visit had mild-to-moderate COVID-19 (*p *= 0.016). The time interval from symptom onset to the first hospital visit was shorter in severely ill children (*p *= 0.013). This finding indicated that children with severe COVID-19 visited the physician more quickly. The prevalence of cough (*p* < 0.001) or stuffy nose (*p *= 0.033) was higher in children with mild-to-moderate COVID-19 than that in children with severe COVID-19. The prevalence of wheezing or dyspnea (*p* < 0.001) and convulsive seizures was higher (*p* < 0.001) in children with severe COVID-19 than in children with mild-to-moderate COVID-19. Correspondingly, the prevalence of severe COVID-19 in children with underlying diseases, such as acute laryngitis (*p *= 0.038), FSs (*p* < 0.001), or epilepsy (*p* < 0.001), was also increased. In comparison, all children complicated with FSs had severe COVID-19 (*p* < 0.001). There were differences in treatment delivery between the two clinical types. Most (76.2%) children with mild-to-moderate COVID-19 took drugs (*p* < 0.001) via the oral route, whereas those with severe COVID-19 (64.7%) mainly had infusions or were hospitalized (*p* < 0.001).

### Characteristics of RBTs in children suffering from COVID-19

Excluding the potential influencing factors of diseases and/or medications on the counts of blood cells, the details of the RBTs of 241 children are summarized in Table 3. For one patient, the parameters related to the mean platelet volume (MPV), platelet-larger cell ratio (P-LCR), platelet crit, and platelet width distribution were absent and were omitted accordingly in statistical analyses. The most common abnormality was an increase in the monocyte count (68.0%). The distribution of abnormal neutrophil count (*p* = 0.048), eosinophil count (*p* = 0.006), mean corpuscular volume (MCV; *p* = 0.033), MPV (*p* = 0.006), P-LCR (*p* = 0.043), and red cell distribution width-standard deviation (RDW-SD; *p* = 0.031) were different in the two clinical types (Table 4). The NLR was significantly increased in the severe type (*p* = 0.003, Table 4). The MLR and PLR were not significantly different between the two types (Table 4).

**Table 3:** Characteristics of routine blood tests in children suffering from COVID-19 without the influence of underlying medical conditions.

Variable	Normal number (%) (1)	High number (%) (2)	Low number (%) (3)	*p*	
				1 vs. 2	1 vs. 3
White blood cells (×10^9^/L)	140 (58.1) 6.26 (5.35–8.06)	29 (12.0) 12.05 (11.12–12.87)	72 (29.9) 5.12 (3.76–6.67)	<0.001	0.004
Red blood cells (×10^12^/L)	76 (31.5) 4.30 (4.14–4.42)	124 (51.5) 4.80 (4.63–4.97)	41 (17.0) 3.74 (3.42–3.83)	<0.001	<0.001
Hemoglobin (g/L)	144 (59.8) 128 (122–132)	34 (14.1) 140 (128–144)	63 (26.1) 109 (102–116)	0.001	<0.001
Platelets (×10^9^/L)	212 (88.0) 205 (172–245)	26 (10.8) 342 (313–375)	3 (1.2) 95 (78–96)	<0.001	0.024
Neutrophils (×10^9^/L)	163 (67.6) 3.37 (2.52–4.28)	41 (17.0) 7.90 (7.05–9.79)	37 (15.4) 1.43 (1.06–1.64)	<0.001	<0.001
Monocytes (×10^9^/L)	76 (31.5) 0.47 (0.38–0.55)	164 (68.0) 0.89 (0.73–1.16)	1 (0.4)	<0.001	1.000
Lymphocytes (×10^9^/L)	121 (50.2) 1.71 (1.34–2.14)	42 (17.4) 4.22 (3.56–5.35)	78 (32.4) 0.84 (0.69–0.95)	<0.001	<0.001
Eosinophils (×10^9^/L)	139 (57.7) 0.09 (0.04–0.16)	2 (0.8) 0.59 (0.51–0.66)	100 (41.5) 0.00 (0.00–0.01)	0.454	<0.001
Basophils (×10^9^/L)	214 (88.8) 0.02 (0.01–0.04)	27 (11.2) 0.09 (0.08–0.13)		<0.001	
Hematocrit (%)	190 (78.8) 38.8 (37.1–40.4)	9 (3.7) 47.0 (46.3–52.2)	42 (17.4) 33.0 (31.5–34.1)	<0.001	<0.001
MCV (fL)	173 (71.8) 85.9 (83.8–88.9)	11 (4.6) 105.4 (101.9–108.1)	57 (23.7) 80.1 (78.5–81.0)	<0.001	<0.001
MCH (pg)	171 (71.0) 28.5 (27.8–29.3)	3 (1.2) 35.0 (34.6–36.4)	67 (27.81) 26.3 (25.4–26.5)	0.096	<0.001
MCHC (gL)	188 (78.0) 331.3 (325.0–336.4)	2 (0.8) 355.4 (355.0–355.8)	51 (21.2) 303.4 (295.8–310.0)	0.166	<0.001
MPV (fL)	199 (82.6) 10.0 (9.5–10.6)	2 (0.8)	39 (16.2) 8.8 (8.5–8.9)	0.124	<0.001
Platelet-large cell ratio	154 (63.9) 25.95 (22.58–29.83)	1 (0.4)	85 (35.3) 15.90 (13.70–17.85)	0.797	<0.001
Plateletcrit	203 (84.2) 0.22 (0.19–0.26)	1 (0.4)	36 (14.9) 0.14 (0.12–0.15)	0.426	<0.001
Platelet distribution width	138 (57.3) 10.7 (10.0–11.8)	3 (1.2) 17.1 (8.1–17.1)	99 (41.1) 8.7 (8.1–9.1)	0.245	<0.001
RDW-CV	207 (85.9) 12.8 (12.4–13.3)	7 (2.9) 16.0 (15.8–17.9)	27 (11.2) 11.7 (11.4–11.8)	<0.001	<0.001
RDW-SD	121 (50.2) 41.3 (40.1–43.6)	6 (2.5) 59.8 (56.0–65.6)	114 (47.3) 37.4 (36.4–38.2)	0.088	<0.001

MCV, mean corpuscular volume; MCHC, mean corpuscular hemoglobin concentration; MCH, mean corpuscular hemoglobin; MPV, mean platelet volume; MLR, monocyte: lymphocyte ratio; PLR, platelet: lymphocyte ratio; NLR, neutrophil: lymphocyte ratio; RDW-CV, red blood cell distribution width-coefficient of variation; RDW-SD, red blood cell distribution width-standard deviation.

**Table 4:** Differences in hematological characteristics among children with different severity of COVID-19 without the effect of underlying medical conditions.

Item	Mild–Moderate			Severe			*p*
	Normal	Increased	Decreased	Normal	Increased	Decreased	
White blood cell count	111 (56.1)	22 (11.1)	65 (32.8)	29 (67.4)	7 (16.3)	7 (16.3)	0.090
Red blood cell	63 (31.8)	97 (49.0)	38 (19.2)	13 (30.2)	27 (62.8)	3 (7.0)	0.109
Hemoglobin	115 (58.1)	28 (14.1)	55 (27.8)	29 (67.4)	6 (14.0)	8 (18.6)	0.437
Platelet count	171 (86.4)	25 (12.6)	2 (1.0)	41 (95.3)	1 (2.3)	1 (2.3)	0.063
Neutrophil count	135 (68.2)	29 (14.6)	34 (17.2)	28 (65.1)	12 (27.9)	3 (7.0)	0.048
Monocyte count	63 (31.8)	134 (67.7)	1 (0.5)	13 (30.2)	30 (69.8)	0 (0.0)	1.000
Lymphocyte count	103 (52.0)	35 (17.7)	60 (30.3)	18 (41.9)	7 (16.3)	18 (41.9)	0.329
Eosinophil count	123 (62.1)	2 (1.0)	73 (36.9)	16 (37.2)	0 (0.0)	27 (62.8)	0.006
Basophil count	173 (87.4)	25 (12.6)		41 (95.3)	2 (4.7)		0.133
Hematocrit	151 (76.3)	9 (4.5)	38 (19.2)	39 (90.7)	0 (0.0)	4 (9.3)	0.114
MCV	146 (73.7)	11 (5.6)	41 (20.7)	27 (62.8)	0 (0.0)	16 (37.2)	0.033
MCH	144 (72.7)	3 (1.5)	51 (25.8)	27 (62.8)	0 (0.0)	16 (37.2)	0.281
MCHC	150 (75.8)	1 (0.5)	47 (23.7)	38 (88.4)	1 (2.3)	4 (9.3)	0.051
MPV	171 (86.4)	1 (0.5)	26 (13.1)	28 (66.6)	1 (2.4)	13 (31.0)	0.006
Platelet-large cell ratio	132 (66.7)	0 (0.0)	66 (33.3)	22 (52.4)	1 (2.4)	19 (45.2)	0.043
Plateletcrit	170 (85.9)	1 (0.5)	27 (13.6)	33 (78.6)	0 (0.0)	9 (21.4)	0.368
Platelet distribution width	117 (59.1)	2 (1.0)	79 (39.9)	21 (50.0)	1 (2.4)	20 (47.6)	0.312
RDW-CV	169 (85.4)	6 (3.0)	23 (11.6)	38 (88.4)	1 (2.3)	4 (9.3)	0.920
RDW-SD	106 (53.5)	6 (3.0)	86 (43.4)	15 (34.9)	0 (0.0)	28 (65.1)	0.031
MLR	0.5450 ± 0.31804			0.6197 ± 0.35089			0.193^a^
PLR	158.3351 ± 101.70923			174.2302 ± 105.09689			0.352^a^
NLR	3.0939 ± 3.54824			4.9484 ± 4.44178			0.003^a^

MCV, mean corpuscular volume; MCHC, mean corpuscular hemoglobin concentration; MCH, mean corpuscular hemoglobin; MPV, mean platelet volume; MLR, monocyte-to-lymphocyte ratio; PLR, platelet-to-lymphocyte ratio; NLR, neutrophil-to-lymphocyte ratio; RDW-CV, red blood cell distribution width-coefficient of variation; RDW-SD, red blood cell distribution width-standard deviation.

^a^ Mann–Whitney test.

### Binary logistic regression analysis of the relationship between hematological factors and COVID-19 severity

We wished to identify the significant hematological factors correlated with COVID-19 severity in children. We conducted a binary logistic regression analysis using variables we found to be significant, along with age and time. The distribution of the MCV, MPV, PLCR, and RDW-SD was significant. There were no significant differences in the distribution of red blood cell (RBC) count and platelet count. Major changes in the proportion of the MPV, PLCR, MCV, and RDW-SD were decreased. Hence, these changes may not be clinically significant. Therefore, the MCV, MPV, PLCR, and RDW-SD were excluded from the binary logistic regression analysis. Finally, we constructed a binary logical regression model of severe COVID-19 in children from the factors obtained (Table 5), including age [odds ratio = 0.847, 95% confidence interval (CI) = 0.736–0.974] as well as decreases in the eosinophil count (2.683, 1.298–5.542) and NLR (1.130, 1.021–1.251). A ROC curve was drawn with the probability value predicted by our regression model. The AUC was 0.749 (95% CI = 0.664–0.834, *p* < 0.001). These data indicated that our model had a certain value in the diagnosis of severe COVID-19 in children.

**Table 5:** Binary logistic regression analysis of the influence of hematological factors on COVID-19 severity in children.

Variable	*p*	Odds ratio (95% CI)
Age	0.020	0.847 (0.736–0.974)
Normal eosinophil count		1
Decrease in the eosinophil count	0.008	2.683 (1.298–5.542)
NLR	0.018	1.130 (1.021–1.251)
Constant	0.00	0.14

NLR, neutrophil: lymphocyte ratio.

### Diagnostic efficacy of RBT items in children with severe COVID-19

**Figure 1:**
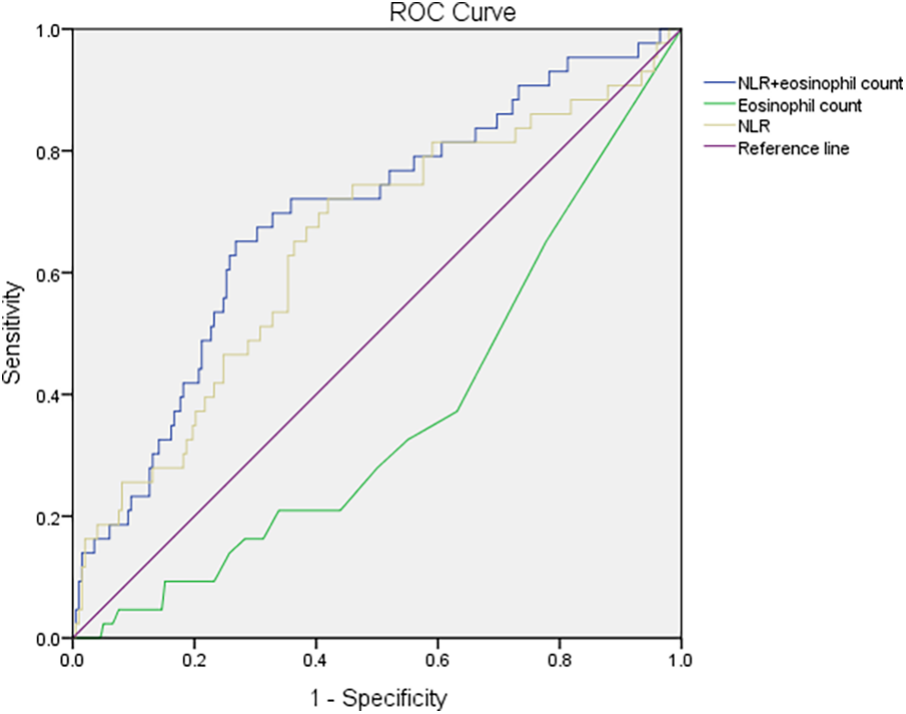
Receiver operating characteristic curves for the NLR, eosinophil count, and NLR combined with the eosinophil count in the diagnosis of severe COVID-19 in children. NLR, neutrophil: lymphocyte ratio; COVID-19, coronavirus disease-2019.

The logistic regression analysis of RBT items revealed significant differences between the mild-to-moderate type and severe type, so ROC analysis was conducted to analyze the NLR and eosinophil count alone or in combination for the diagnosis of severe COVID-19 in children. The AUC of the NLR was 0.643 (95% CI = 0.548–0.739, *p *= 0.003). The AUC of the eosinophil count was 0.366 (95% CI = 0.277–0.455, *p *= 0.006). The AUC of the NLR combined with the eosinophil count was 0.688 (95% CI = 0.599–0.777, *p* < 0.001) (Table 6). The combination of the NLR and eosinophil count presented the largest AUC. The maximum Youden Index was 0.3835, and the associated criterion was >0.1983. The sensitivity and specificity were 65.1% and 73.2% respectively, Correspondingly, the NLR >2.17 and the eosinophil count in routine blood tests decreasing approximately to zero might have indicated severe infection in children suffering from COVID-19. The ROC curve is shown in Figure 1. Further analysis was done to estimate the diagnostic value of the NLR combined with the eosinophil count in different age groups of children. The AUC increased with a decrease in age (Table 6).

**Table 6:** Benefit of using the NLR combined with the eosinophil count in the diagnosis of severe COVID-19 in children of different age groups.

Group	AUC	*p*	95% CI
0–14 years	0.688	<0.001	0.599–0.777
<6 years	0.717	<0.001	0.622–0.812
<3 years	0.821	<0.001	0.735–0.907
<1 year	0.833	0.027	0.685–0.982

## Discussion

We analyzed the differences in clinical and RBT characteristics between children with mild-to-moderate COVID-19 and severe COVID-19. Young age, a decreased eosinophil count, and increased NLR at the initial period of SARS-CoV-2 infection were risk factors for severe COVID-19. We also revealed that the combination of the NLR and eosinophil count might be an effective indicator for the diagnosis of severe COVID-19. Taken together, these results may help clinicians identify children with severe COVID-19 so that they can undertake treatment on time to improve the outcomes for children infected with the Omicron or new variants. Our findings may also provide a scientific reference for formulating targeted management strategies and optimizing the allocation of medical resources in COVID-19 epidemics in the future.

The median age of the population was 2.0 (IQR, 0.9–6.0) years, which is similar to results from other studies (8, 14) after the Omicron variant had been detected (hereafter termed “the Omicron period”), and confirmed that children infected with the Omicron variant were younger than children infected with other variants of SARS-CoV-2. We revealed that the distribution of COVID-19 severity in children of different age groups was significantly different and that young age was a risk factor for severe infection with the Omicron variant in children. However, this association must be considered with caution. In our study cohort, 50% were younger than 3 years of age, and they were not covered by vaccination against SARS-CoV-2. Moreover, data focusing on the relationship between age and COVID-19 severity in children are scarce. However, one systematic review (15) showed that the ratio of severe or critical infection in those aged <1 year was higher than that in older children. The anatomic characteristics of a relatively narrow respiratory tract and immunological characteristics of children who have not been vaccinated could be reasons for those data.

Due to the inconsistent use of diagnostic criteria for COVID-19 severity in different studies, direct comparison of the prevalence of COVID-19 severity in different investigations is difficult. The prevalence of the severe type of COVID-19 in our study, 19.54%, is similar to that in the Omicron period reported by Choi and colleagues (14) from South Korea, who used the same diagnostic criteria. Han et al. (16) and Li et al. (17) reported a similar prevalence (0.7%) in their study populations. Wang et al. (18) did not report any severe cases of COVID-19 in their study. Overall, the prevalence of severe COVID-19 in our study was higher than that revealed by other studies in the Omicron period. We attribute this difference to four main factors. First, the study population was different. Our patients were symptomatic, whereas those in the studies of Wang et al. (18) and Li et al. (17) were asymptomatic. Second, Second Affiliated Hospital of Xi'an Jiaotong University is a tertiary hospital, with abundant medical resources. If parents seek treatment, they will send their seriously ill children to a tertiary-level hospital. Third, our study and other studies have revealed that childhood COVID-19 is characterized by a higher prevalence of FSs and laryngitis. Fourth, as stated above, our study cohort had a higher proportion of children under the age of 3 years, who were not vaccinated against SARS-CoV-2 and, therefore, had more severe COVID-19 (19).

Consistent with the characteristics of SARS-CoV-2 variants documented by other scholars and in systematic reviews (15), fever and cough were the most prevalent symptoms at the time of the COVID-19 diagnosis. In addition, the prevalence of convulsions (7, 14, 20) and hoarseness (14) was high in children infected with the Omicron variant in the present study and other studies. Specific symptoms described in adults (21), such as dysgeusia, were not found in children in the present study. This disparity may (16) relate to the underdeveloped language skills of most young children, who cannot express their feelings directly. Besides the diagnosis of COVID-19, acute infection of the upper respiratory tract was the most common diagnosis. The prevalence of FSs and croup was also high compared with that in previous studies (14, 16) in which different SARS-CoV-2 variants predominated. Joung et al. (20) described the features of FSs associated the children infected with the Omicron variant in Korea. Their study cohort showed a longer duration and greater number of seizures, a higher prevalence of complex FSs, along with a higher mean age and higher peak body temperature of children. Therefore, recognition and management of FSs and croup by the carers of children suffering from COVID-19 are very important.

Monocytes have multiple roles in various pathologic conditions, including the inflammatory response after infection. In most patients infected with SARS-CoV-2, the monocyte count in peripheral blood is increased (22, 23) and we noted a significant increase in the monocyte count in most of our patients. This increased monocyte count may be an attempt to suppress replication of SARS-CoV-2 viruses, thereby reducing the severity of COVID-19. Analyses of circulating monocytes have also been shown to predict COVID-19 severity (24). However, we did not find that the monocyte count could be used to predict COVID-19 severity in children, and this may have been due to the small number of patients with severe COVID-19.

Eosinophils, as innate immune cells, are traditionally associated with allergic and parasitic diseases (25). However, recent studies have suggested that eosinophils could play functions in viral infections (26–31), and might be potential biomarkers in respiratory viral infections (31). The functions have also been found in COVID-19 infections (31, 32). Additionally, SARS-CoV-2 receptor ACE2 is expressed less in the lung tissue of asthmatic patients (33) and the eosinophilic nasal polyp tissue (34). As a result, allergic persons have less risk of having or developing severe COVID-19 (33, 34). In studies in adults with COVID-19, the eosinophil count was reduced in patients with acute respiratory deterioration (35), and the reduction was more common in moderate and severe types of COVID-19 (36). Patients suffering from COVID-19 with a lower eosinophil count had a lower chance of survival (36). In addition, the eosinophil count was reduced in the initial phase of COVID-19, and increased gradually to the normal range upon recovery from COVID-19, thereby suggesting the role of eosinophils as indicators for disease progression and treatment efficacy (36). In pediatric patients with COVID-19, Arikan and colleagues (23) found that the absolute count of eosinophils upon hospital admission was lower than that for healthy children, and that the eosinophil: monocyte ratio might be useful in the diagnosis of pediatric COVID-19. Du and colleagues (37) stated that 29.5% of children with COVID-19 experienced eosinopenia upon hospital admission. In our study, 41.5% of pediatric patients had eosinopenia, and the prevalence of eosinopenia was significantly different in different types of COVID-19. These findings suggest that the eosinophil count might be a promising hematological parameter for identifying or evaluating the status of COVID-19 in zchildren.

Neutrophils, as part of the first line of innate immune defense, are crucial for the host response to viral infections (38). However, in SARS-CoV-2 infection, excessive numbers of neutrophils can result in a “storm” of proinflammatory cytokines (“cytokine storm”) and tissue injury due to oxidative burst, phagocytosis, and the formation of neutrophil extracellular traps, thereby aggravating COVID-19 severity (39). A growing body of evidence suggests that the neutrophil count is increased in the peripheral blood of patients with severe COVID-19 (40) and patients who died from COVID-19 (41). Wang et al. (39) demonstrated that the dynamics of the neutrophil count in hospitalized patients with COVID-19 exhibited the same trend as the corresponding lung injury, which suggested that neutrophilia-mediated inflammation had an important role in the pathology and severity of COVID-19. The NLR, being a ratio, is a more reliable indicator than the neutrophil count alone and is associated with the cytokine storm in COVID-19 (42, 43), which can be used to predict COVID-19 severity (44). However, few studies have focused on the role of the NLR in children with COVID-19.

Alshengeti and coworkers (45) reported that pediatric patients with severe illness had a higher NLR than those with milder disease, but the difference was not significant. Gizem and colleagues (46) reported the NLR in children with confirmed COVID-19, severe COVID-19, and hospitalized children to be higher than that in children with suspected COVID-19, children with non-severe COVID-19, and children being treated as outpatients, respectively, and that the increase in the NLR was not significant. The NLR varies in different phases of COVID-19, but studies have not adjusted for the influence of the time point of RBTs on the NLR. Arikan and collaborators (23) demonstrated that the NLR was increased significantly in children with confirmed COVID-19 compared with that in healthy children. We discovered that the prevalence of neutrophilia and the NLR was higher in children with severe COVID-19, and further analysis confirmed the NLR to be a risk factor for severe COVID-19. In our study, RBTs were carried out at the first hospital visit, and the NLR in the regression model was adjusted by the time interval from symptom onset to the first hospital visit, which made our results more rigorous. Therefore, the NLR might be another indicator for assessing COVID-19 severity in children.

In the present study, the combination of the NLR and eosinophil count showed the largest AUC, indicating that this combination was the most discriminative hematological parameter for a clinical diagnosis of severe COVID-19 in children. Specifically, the NLR >2.17 and the eosinophil count in routine blood tests decreasing approximately to zero might have indicated severe infection in children suffering from COVID-19. At this point, a clinician should attach more attention and take positive strategies to manage COVID-19 in children. Analyses by specific age groups revealed the discriminative power of this combination to increase with younger age, and the AUC was ≤0.833 in children younger than 1 year. These data suggested that a combination of the NLR and eosinophil count might be a reliable parameter for pediatricians recognizing severe COVID-19 in children at an early stage. The MLR and PLR have also been reported to be useful in monitoring COVID-19 in adults (47), but we did not find that in our pediatric study.

Our study had three main limitations. First, our cross-sectional, retrospective, single-center study with a small sample size limited the generalizability of our findings. Second, other respiratory pathogens were not tested in all subjects. Third, the inconsistency of symptoms reported by the carers of young children and those described by older children may have led to some bias.

## Conclusions

Most of the children in our study had mild-to-moderate COVID-19. The prevalence of FSs and croup was increased during the Omicron period. Abnormalities in the NLR and eosinophil count at the onset of SARS-CoV-2 infection were risk factors for COVID-19. The combination of the NLR and eosinophil count might be a promising indicator for identifying severe COVID-19 at an early stage. Our findings provide a reference for precise management of COVID-19 in children in future SARS-CoV-2 epidemics.

## Data Availability

The raw data supporting the conclusions of this article will be made available by the authors, without undue reservation.
